# A conversation on the impacts and mitigation of air pollution

**DOI:** 10.1038/s41467-021-25518-2

**Published:** 2021-10-04

## Abstract

Air pollution is an environmental and health concern affecting millions globally every day. Dr Audrey de Nazelle, an expert in air pollution risk assessment and exposure science at Imperial College London, shares with *Nature Communications* their thoughts on the impacts of air pollution and the policies needed to tackle emissions.

**Figure:**
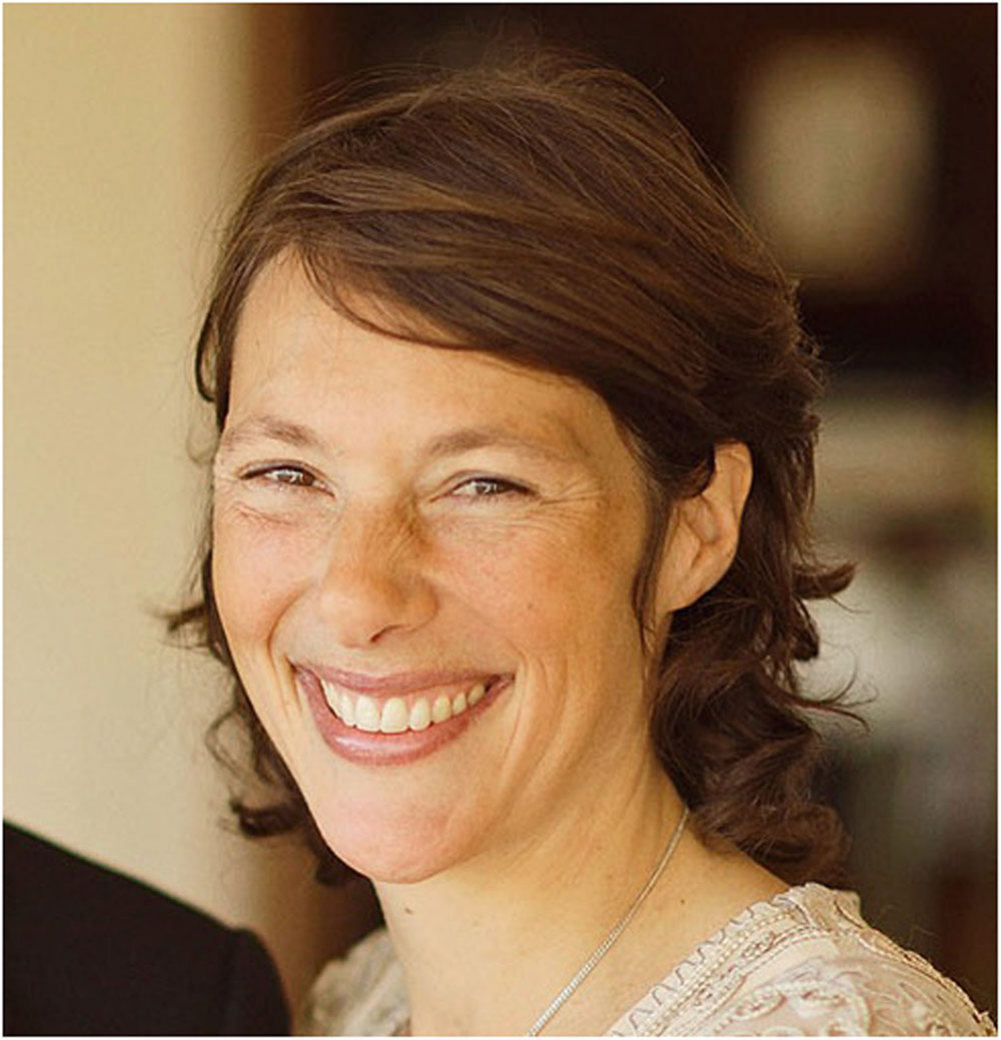
Audrey de Nazelle.

What aspect of air pollution concerns you the most?

Air pollution is detrimental to our health at every stage of our lives, affecting almost every organ of our bodies, and most people can do little to limit their exposures. There are no known safe levels of ambient air pollutants we encounter in our daily lives, such as particulate matter and nitrogen dioxide. As a society, we would benefit from everyone reducing their exposures, with population health benefits ranging from reproduction and neonatal outcomes, lung and cognitive development, respiratory and cardiovascular health, diabetes and obesity prevention, and protection against infectious diseases. It would particularly benefit deprived populations—low income and minority ethnic groups tend to have both greater exposures and greater susceptibility to adverse health outcomes related to air pollution than more advantaged populations. Individuals alone, however, have only limited power to protect themselves from the ill effects of air pollution; achieving reductions in population exposures to air pollution requires bold policies and collective action.

If well-devised, such bold air pollution policies additionally provide the opportunity to bring further health benefits beyond those accrued from air pollution reductions alone. In cities, for example, where typically the largest fraction of ambient pollutants stem from transport, ambitious policies that create environments conducive to walking, cycling or taking public transport instead of driving will also bring about healthy levels of physical activity, lower traffic injuries, or more room for green and open space.

Most individuals are helpless in the face of harmful and inequitable exposures, and it is the lack of widespread recognition of both the *opportunity* and *responsibility* to improve the health and equity of our society through collective action that concerns me the most about air pollution. In my research, I focus on the opportunity provided by urban transformations to promote healthy, sustainable, and equitable environments.

What are your thoughts on current policy enforcement, and how well or not this is being achieved?

More than enforcement of policies, what is needed is bolder policies. Air pollution standards are lax in most areas of the world—air pollution impacts occur far below the European 25 µg/m^3^ limit value for example, and even below the WHO’s current guideline of 10 µg/m^3^. The formulation of standards is also inadequate to achieve widespread population health: areas that are currently in compliance with regulatory standards have no incentive to further reduce air pollution, even though we know we could achieve further health benefits by shifting the entire distribution of population exposures towards lower concentrations. Policies that are put in place to achieve standards are often near-sighted and narrow-minded. The lack of joined up thinking across policy sectors inhibits the kind of holistic vision needed for efficient policy making that truly delivers on the promise of air pollution policies, i.e., to promote health and wellbeing. Policy frameworks that require systemic thinking are needed so that feedback effects and multiple outcomes of decisions are accounted for.

How effective is voluntary action vs government mandated policy in reducing air pollution?

Voluntary action and government mandated policies go hand in hand. Government action is needed to enable and empower individuals to make sustainable and healthy choices. Taking the example of urban environments again, getting people out of their cars ultimately requires bold action on the part of governments to make public transport, walking, and cycling be the easy and most appealing choices for all. This means transforming the urban landscape so that people live close to their everyday destinations, and so that streets are safe and comfortable to enjoy cycling and walking in—even for families with children. It also means investing in cities so they are places worth living in rather than escaping from at every opportunity one could afford. Government actions to ensure affordable living conditions are ensured for all to reap the benefits of the healthy urban transformations is also key.

In reverse, buy-in and support from city dwellers and local stakeholders are required to embolden policymakers towards such transformative actions. Making multiple and far-reaching trade-offs and benefits salient in the decision making will help engage citizens and create the partnerships that enable effective action towards desired visions of city landscapes.

Socioeconomic factors such as income, education and wealth have been shown to play a key role in public health air pollution impacts. What needs to be done to ensure that policies developed are equitable and just?

Deprived populations suffer the most from air pollution, and typically contribute the least to air pollution in cities. Individuals of lower socio-economic status have lower car ownership rates and drive less than more advantaged populations. Yet, car reduction strategies are often opposed on the grounds of being most unfair to the poor. Controversial low traffic neighbourhoods in London are a case in point, though research has shown they have so far been deployed in majority in streets housing populations in lower deciles of deprivation. It is of course possible that such traffic reduction schemes displace traffic onto surrounding major roads where lower income people may live. The same way, however, that road building eventually leads to more car travel, reducing space given to cars eventually reduces the amount of traffic, although there may be a period of adaptation needed for the new equilibrium to be reached. The key is of course to ensure alternatives to car use are attractive and affordable to all.

Gentrification is a real concern for regeneration projects that make communities more conducive to walking and cycling. By attracting wealthier newcomers, creating more human scale neighbourhoods can indeed end up displacing or marginalising existing populations. At the local level, it is essential to foster citizen participation in the planning development process to ensure adequate options for affordable living conditions (housing, shopping, public transport options) are maintained. More importantly in the long run, widespread adoption of people-friendly environments across the city landscape will make each individual pocket less prized by the wealthier populations and hence limit gentrification processes.

More generally, engraining equity goals across policy areas will ensure joined up thinking and create alliances across groups and sectors for the promotion of healthy, sustainable, and equitable societies.

Technological advances to mitigate air pollution such as retrofitting coal-fired plants are touted as potential cost-effective solutions. What are the most promising recent advances to mitigate against pollutants?

Technological solutions are part of the solution. In the city context, for example electric or hydrogen vehicles have their place in the portfolio of actions needed to tackle air pollution. They are needed to bring down emissions from buses, ambulances, delivery trucks, or other service vehicles that are required for the good functioning of society. Communication and sensing technology also hold some potential in the fight against air pollution. Travel apps have made scheduling of public transport use far more tractable, and facilitated way finding for pedestrians and cyclists. Air pollution-related apps and sensors have the potential to engage citizens towards protective behaviours to minimize exposures, towards mitigating behaviours to reduce contributions to air pollution, and towards policy support for ambitious policies. The evidence base on the effectiveness of such approaches however is limited, in part because apps assessed so far have not been designed to fully integrate learnings from air pollution communication research (e.g. integrating a full range of actionable information or fostering collective action).

Do you hold out more hope for technological solutions, or political action, as a means to reduce air pollution?

Technological solutions to address air pollution are often low-hanging fruits to gain relatively quick and painless wins. They typically offer no co-benefits, are rarely transformative and can be loaded with trade-offs or unintended consequences that can eventually back-fire. They have their place in the multitude of efforts required to bring down noxious levels of air pollution—for example electrically-powered ambulances or bin collection vehicles. Single-minded focus or over-reliance on technology, however, is at best a wasted opportunity for further co-benefits, and at worst creates a lock-in into a system that prevents further gains in the long run. Electrification of the vehicle fleet, for example, requires large investments from local authorities to create an adequate charging network, and individual private investments to buy new cars. Such investments present an opportunity cost for funding that could otherwise be used for more radical healthy urban transformations, and can create inertias that prevent these more fundamental changes from taking place. In addition, its benefits on air pollution are only limited as electric vehicles continue to emit particles from tyre and brake wear (currently the large majority of particulates emitted from cars). It partly just displaces emissions as electricity still needs to be produced to power the vehicles. It generates health hazards in poor populations of low-income countries where rare metals are mined to make batteries. It perpetuates ailments of car-oriented societies including large health burden from traffic hazards and sedentary lifestyles. Bold political actions to push cities to be less car-reliant, on the other hand, can help create resilient, healthy, and sustainable cities people want to live, work, and play in.

Finally, how would you like collaboration between physical, health and policy scientists working on air pollution to improve?

Transformative solutions to air pollution, especially in the context of urban change towards people-friendly, human-scale, sustainable, equitable and healthy environments, will require concerted efforts across sectors, including academic disciplines. One of the greatest hindrances towards such radical changes is the lack of political will or leadership. From an academic standpoint, what is needed is to evaluate decision-making processes to identify leverage points and to develop a convincing evidence-base for optimal solutions. This requires collaborations across disciplines from social to physical sciences to understand the inter-linkages between urban form, behaviour change, political processes, environmental phenomena and health and social impacts. Research outputs, however, are often considered irrelevant to decision makers who may view their own contexts as overly complex and unique. To ensure such research developments are grounded in real policy contexts and produce knowledge that is both useful and used, academics can strive to develop their research programmes in partnership with a range of relevant stakeholders, including policymakers. Co-created research helps academics along every step of the way to have maximal impact- from posing the right questions, to choosing research outputs that rings true in the policy decision making context, and finally translating and disseminating the research so it is understood and heard in relevant policy settings. Applying systems thinking in research development will also help capture the complexity of real-world phenomena, and identify interlinkages, feedback effects, trade-offs, and co-benefits in the decision make process. Collaborations across disciplines in the context of air pollution policy making could thus be greatly improved by encouraging academics to co-create knowledge and solutions and applying systems thinking in research development.

*This interview was conducted by Melissa Plail.*

